# Circulating biomarkers of vasoplegia: a systematic review

**DOI:** 10.1186/s13613-025-01564-7

**Published:** 2025-09-30

**Authors:** Naomi Boyer, Prateek Upadhyay, Megan H. Hicks, Alexander Zarbock, Ashish K. Khanna, Lui G. Forni, Benedict C. Creagh-Brown

**Affiliations:** 1 Intensive Care Unit, Royal Surrey NHS Foundation Trust, Guildford, UK; 2 School of Biosciences, University of Surrey, Surrey, UK; 3 Smidt Heart Institute, Cedars-Sinai Medical Center, Los Angeles, CA USA; 4 Department of Anesthesiology, Section on Critical Care Medicine, Atrium Health Wake Forest Baptist Medical Center, Wake Forest University School of Medicine, Winston-Salem, USA; 5 Department of Anesthesiology, Intensive Care and Pain Medicine, University Hospital Münster, Münster, Germany; 6 School of Medicine, University of Surrey, Surrey, UK

**Keywords:** Vasoplegia, Hypotension, Septic shock, Distributive shock, Vasopressors, Vascular tone, Biomarkers

## Abstract

**Background:**

Vasoplegia is characterised by persistent hypotension and reduced systemic vascular resistance despite preserved cardiac output, commonly arising in sepsis, following major surgery, and within systemic inflammatory responses. Despite its clinical significance and association with poor outcomes, there is no universally accepted definition or standardised biomarker, impeding early diagnosis, stratification, and targeted therapy. While individual studies have examined biomarkers within specific clinical contexts such as septic shock or cardiac surgery, no comprehensive synthesis across all aetiologies of vasoplegia has previously been undertaken.

**Objectives:**

To systematically evaluate and synthesise the current evidence regarding circulating biomarkers associated with the incidence, severity, prediction, and progression of vasoplegia across diverse critical care and perioperative populations. As well as review definitions used across literature.

**Methods:**

This systematic review was conducted in accordance with PRISMA 2020 guidelines and registered on PROSPERO (CRD42024438786). Studies were included if they investigated adult patients in critical care or perioperative settings with vasoplegia defined by reduced vascular resistance and hypotension requiring vasopressors.

**Results:**

A total of 43 studies met inclusion criteria. The included studies examined 39 unique biomarkers, with renin and adrenomedullin being the most frequently studied. Heterogeneity in definitions of vasoplegia, outcome measures, and comparator populations precluded meta-analysis. However, several biomarkers demonstrated potential clinical utility: elevated renin levels correlated with vasopressor requirements and haemodynamic instability, while adrenomedullin levels were predictive of vasoplegia development and duration.

**Conclusions:**

The lack of standardisation in biomarker assay methods and vasoplegia definitions remains a significant barrier to comparative analysis. Whilst this review highlights renin and adrenomedullin as promising candidate biomarkers for vasoplegia, the heterogeneity in study design, biomarker measurement, and diagnostic criteria underscores the urgent need for a consensus definition of vasoplegia, standardised sampling protocols, and unified outcome measures. Future research should focus on biomarker-guided risk stratification and personalised therapies, with an emphasis on validating predictive and mechanistic roles across diverse vasoplegic phenotypes.

**Supplementary Information:**

The online version contains supplementary material available at 10.1186/s13613-025-01564-7.

## Background

### Context

Vasoplegia describes a phenomenon characterized by profoundly reduced vascular tone that may manifest with hypotension despite preserved or elevated cardiac output [1]. Severe cases may progress to vasoplegic shock (often used interchangeably with distributive shock), which is distinguished by tissue hypoperfusion and frequently accompanied by hyperlactatemia [2, 3]. This condition has causes including sepsis, anaphylaxis, pancreatitis, burns, and major cardiac and non-cardiac surgery. Independent of the precipitating cause, vasoplegia is associated with poorer outcomes, including increased mortality [4], higher incidence of acute kidney injury, and extended ICU and hospital stays [5–7].

### Current knowledge

Different aetiologies (subphenotypes) of vasoplegia share both similarities and differences in their underlying mechanisms. For instance, in pancreatitis, burns, and post-surgical states, tissue injury triggers the release of damage-associated molecular patterns (DAMPs), which activate the systemic inflammatory response, resulting in comparable clinical presentations across these diverse precipitants. Similarly, sepsis involves pathogen-associated molecular patterns (PAMPs) that elicit systemic inflammation. In contrast, anaphylactic vasoplegia occurs in response to the release of specific vasoactive mediators, suggesting a potentially distinct pathophysiological pathway.

Currently, there exists no standard definition of vasoplegia or vasoplegic shock, nor have any documented attempts at establishing an expert consensus definition been reported. Diagnosis is typically made on clinical grounds, often without comprehensive haemodynamic data from cardiac output monitoring. There is no established assay of any circulating biomarker to assist in the diagnosis, characterisation or management of vasoplegia, although numerous studies have investigated biomarkers of vasoplegia in specific contexts such as septic shock or post-cardiac surgery.

### Knowledge gap

A comprehensive review that synthesizes biomarker profiles across all vasoplegic states remains absent from the literature. A robust, accessible and easily quantifiable biomarker for vasoplegia could offer a range of potential benefits. Firstly, a biomarker that could indicate a propensity to develop vasoplegia, for example following a known precipitant such as elective surgery or early sepsis, could assist risk stratification to aid early decision-making regarding optimal treatment environment. Secondly, identifying a superior response to a non-catecholamine vasopressor would influence treatment and improve outcome. Thirdly, insights into the pathophysiological mechanisms may allow subphenotypes to be considered endotypes (a subtype of a disease that is defined by a specific biological mechanism that causes the disease’s characteristics), indicating the possible need for an alternative therapeutic approach.

### Importance statement

Identifying reliable circulating biomarkers for vasoplegia is critical for improving patient outcomes through earlier risk stratification, more targeted therapeutic approaches, and enhanced understanding of underlying pathophysiological mechanisms across different clinical contexts.

### Objective

This systematic review aimed to systematically assess the current state knowledge regarding biomarkers which relate to the entire spectrum of vasoplegia within critical care and perioperative populations.

## Methods

The primary aim of this systematic review was to ascertain the current knowledge regarding biomarkers relating to vasoplegia within the critical care population. We also aimed to capture how vasoplegia was being defined across the literature. It followed the Preferred Reporting Items for Systematic Reviews and Meta-analyses (PRISMA) 2020 guidelines [1] and was registered with the PROSPERO database (CRD42024438786; registration date 28/03/2024). We included all study types published in English language journals, with no date restrictions, involving adult critical care populations experiencing vasoplegia. The studies needed to relate a biomarker to the incidence, predictive capabilities, severity, or time course of vasoplegia.

### Inclusion/exclusion criteria

Given the lack of consensus definition of vasoplegia we used pre-agreed criteria to select articles where vasoplegia was investigated. For vasoplegia to be identified there needed to be evidence of compatible cardiac output derived variables (reduced systemic vascular resistance with preserved cardiac output), hypotension requiring vasopressors, or persistent hypotension despite fluid resuscitation.

Studies were excluded if shock was due to cardiogenic, obstructive, traumatic, or hypovolaemic causes. Additionally, patients receiving extracorporeal membrane oxygenation (ECMO) or mechanical circulatory support were also excluded.

### Search strategy and paper selection

A comprehensive search strategy developed with librarian assistance of Embase, Medline, Ovid, Web of Science and the Cochrane Library was conducted. The complete search protocol is available in Appendix 5. Given the limitations of the search strategy, including the fact that “vasoplegia” only became a MeSH term in 2016, we also reviewed and utilised citations from papers identified through the search protocol that could not be directly included.

Paper titles were initially screened by NB. Subsequently, all abstracts were independently reviewed by two authors (NB and PU). In cases of disagreement, a third independent party (BCB) was consulted to adjudicate. Full texts of the selected abstracts were then independently reviewed by the same two authors (NB and PU). Any disagreements during the full text review were referred to a third author (BCB) for resolution.

### Outcome data collection, extraction and analysis

Two authors (NP and PU) reviewed and extracted data from the included manuscripts in a data collection form that had been standardised. Risk of bias assessment was performed using the Quality Assessment Tool for Diagnostic Accuracy Studies (QUADAS-2) [2] across the four key domains of patient selection, index test, reference standard and flow and timing. This was assessed independently by NB and PU and was characterised as low risk, high risk or unclear.

### Biomarkers

As the primary focus of the review was circulating biomarkers and their association with the incidence, severity or duration of vasoplegia we collected data on types and quantities of biomarkers reviewed, numerical data on changes in biomarker levels over specific time frames, and biomarker thresholds for diagnosing vasoplegia, as well as statistical methods used by each individual study for their associations. The timing of the measurement of the biomarkers being studied did not impact on inclusion or exclusion. Studies were excluded if the biomarker was not related to the extent of shock or vasoplegia incidence or duration.

### Vasoplegia definitions

In the absence of a consensus definition, we aimed to collate and summarise the different approaches investigators have used to define vasoplegia. The following approaches were acceptable for inclusion in this review. We collected how each individual study defined vasoplegia and where included noted individual thresholds for vasoplegia diagnosis.

### Data analysis

Given the unknown quantity and breath of studies relating to biomarkers of vasoplegia, it was decided that if a sufficient number of studies (at least three) were addressing the same biomarker with the same outcome measures, then a meta-analysis would be conducted, otherwise we would provide a narrative summary of the results.

## Results

The initial search identified 24,174 records. Following manual and computer assisted removal of duplicates and exclusions based on title, 1314 abstracts were reviewed, leading to a further 1177 papers being excluded. 137 manuscripts were included for full text review, with 43 being included in the main analysis. Full texts were excluded primarily because they did not include vasoplegia or a related surrogate as primary or secondary outcomes, or the biomarker studied was unrelated to vasoplegia. This is summarised in the PRISMA flow chart in Appendix 1.

### Study characteristics

Most studies were prospective observational cohort studies (*n* = 23, 53.4%). Interventional studies were rare, with only two being included in this review [3, 4]. Five studies conducted *post hoc* analysis of larger observational or interventional studies [5–8]. The majority of studies (58.1%) were of septic patients; the post cardiothoracic surgery cohort was the next most common with 15 studies (14 of which were following cardiac surgery [3, 6, 9–20] with the remainder following thoracic surgery, specifically lung transplantation [21]). Three studies included vasodilatory shock of mixed aetiology [5, 22, 23]. Figures summarising this can be found in the supplementary online document.

The choice of comparator population varied significantly between studies. Several (*n* = 6, 14.0%) opted for no control group [3, 22, 24–27], whereas nine studies had healthy controls [5, 23, 28–34]. By contrast, four studies chose multiple controls, whereby they included healthy controls, as well as the population of interest who did not experience vasoplegia (e.g. septic patients or those following cardiothoracic surgery, but not meeting criteria for shock/vasoplegia) and healthy controls [9, 34–36]. These differences in comparator groups made comparison between the studies of the same biomarkers challenging as often there was no overlap of outcome measures or control groups.

The QUADAS-2 [2] risk of bias assessment was performed for all studies. The highest area of bias susceptibility was found within patient selection, with only 16 studies having a low risk assessment. The remaining were either high risk (*n* = 15) or unclear (*n* = 12). The other categories within QUADAS-2 assessment (index test, reference standard and flow and timing) had few studies scoring high risk. A figure summarising this can be found in supplementary document, appendix 2.

### Biomarkers

Of the 43 studies included in this review, a total of 39 different biomarkers were identified. Several studies examined two or more biomarkers. Renin and adrenomedullin were the most common biomarkers investigated with five studies investigating each molecule, heparin binding protein and IL-6 were the next most common biomarker studied, with four different studies investigating each biomarker.

**Fig. 1:**
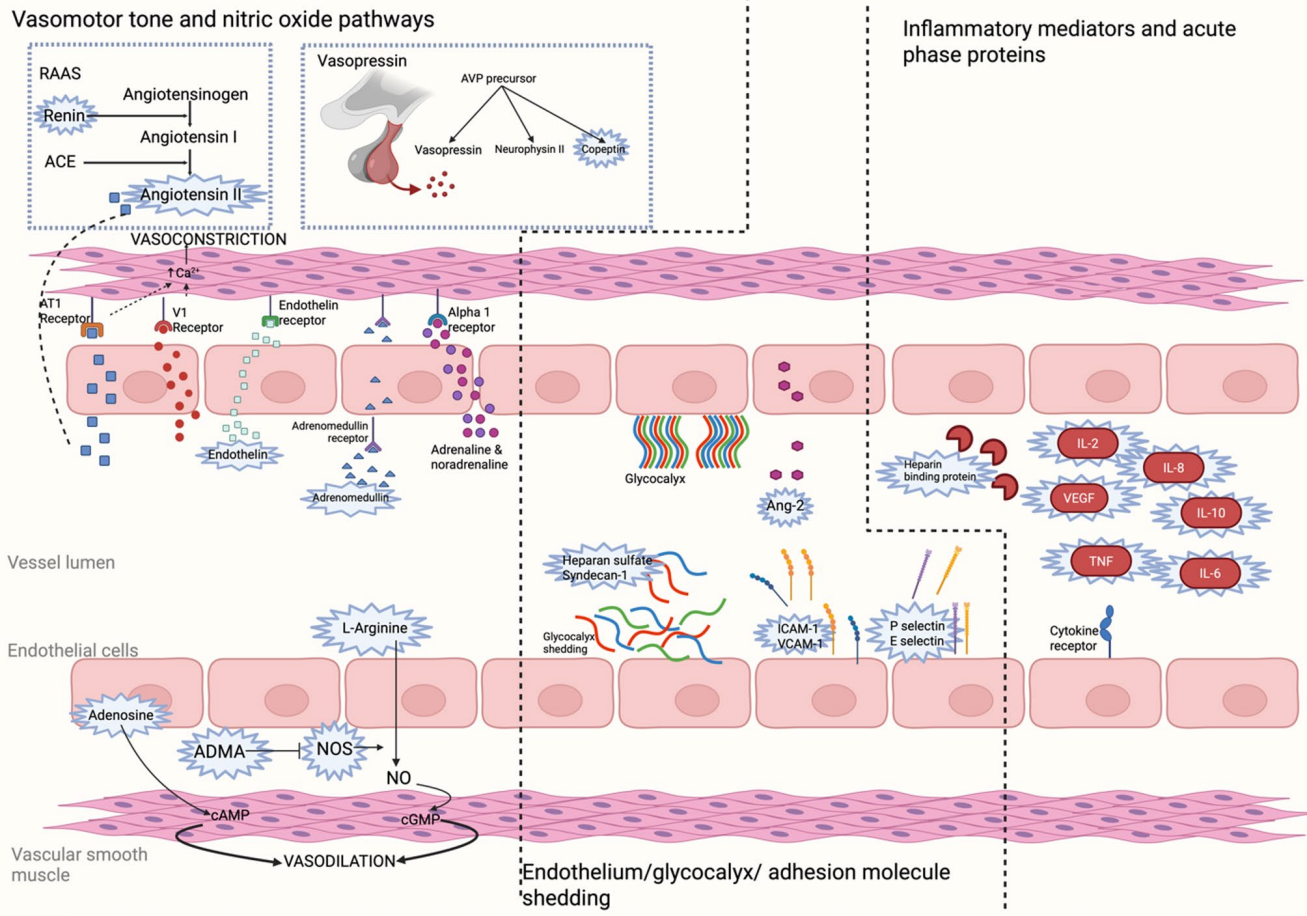
Diagrammatic representation of biomarkers and mechanisms involved in vasoplegia (biorender.com). Biomarkers covered in this review are highlighted in blue. The adrenergic system plays a crucial role within vasoconstriction with both adrenaline and noradrenaline acting on alpha 1 adrenoreceptors on the smooth muscle cells, activation of which ultimately results in increased intracellular calcium and smooth muscle contraction. The renin angiotensin aldosterone system (RAAS), vasopressin, endothelin and adrenomedullin all play a role in the maintenance of vasomotor tone. Angiotensin II and vasopressin bind and activate their receptors located within the endothelial and vascular smooth muscle cells. Activation of these receptors results in increases in cytosolic calcium, causing phosphorylation of the myosin light chain, leading to actin-myosin interactions, and ultimately resulting in smooth muscle contraction. Dipeptidyl peptidase 3 (DPP3) is an enzyme involved in the degradation of multiple peptides within RAAs including angiotensin I and II. Arginine vasopressin (AVP) is also an indirect vasoconstrictor through inhibition of nitric oxide production and inducing the opening of ATP dependent K + channels. Copeptin is derived from the precursor of AVP and can be used as a surrogate of the concentration of AVP, it does not act directly on the vascular endothelium or smooth muscle cells. Adrenomedullin and endothelin are released from endothelial cells in response to inflammatory cytokines and shear stress, acting on receptors located on vascular smooth muscle. Endothelin receptor activation results in increase in cytosolic calcium via IP3 and DAG. Adrenomedullin also causes increases in intracellular calcium and associated smooth muscle contraction. Nitric oxide (NO), produced from l-arginine via nitric oxide synthase (NOS) is a key component in vasodilation, asymmetric dimethylarginine (ADMA) inhibits the production of NO. Adenosine is another potent vasodilator acting on receptors within the vascular smooth muscle cells. In response to shear stress, interactions with inflammatory mediators and trauma, alterations to the endothelial glycocalyx occur, resulting in vascular inflammation and endothelial dysfunction. Heparan sulfate and syndecan-1 are both components that are shed and have been used as biomarkers in several studies. Multiple inflammatory mediators have been implicated in studies, these include TNF and IL-6 the canonical pro-inflammatory cytokine that is secreted predominantly from macrophages and monocytes in response to PAMPS. Heparin binding protein is released predominantly from activated neutrophils in multiple inflammatory states and has multiple effects, including alterations to endothelial function such as increased permeability. Created in BioRender. Boyer, N. (2025) https://BioRender.com/helwi5i

The intention was to undertake a meta-analysis review relating the measured biomarkers with aspects of vasoplegia. However, this was impossible to undertake due to a lack of uniform outcome measure between studies, heterogeneous populations, and lack of common definitions. Therefore, the biomarkers have been grouped by mechanism and each will be discussed in a narrative synthesis, Fig. 1 is a diagrammatic representation of the different mechanisms. Table 1 represents the diversity of biomarkers (grouped by general mechanism or origin) and shows how many studies were focusing on each biomarker. Appendix 3 contains a detailed summary of all 43 studies and all the biomarkers included within this review.

Only 20 of the 43 studies had the biomarker of investigation being associated with vasoplegia (*n* = 13) or biomarker for prediction of vasoplegia (*n* = 7) as a primary outcome measure of the study. The most common primary outcome measure was examining the association of the biomarker with clinical outcomes or illness severity (*n* = 10 for outcomes, *n* = 8 for illness severity), with the next most common primary objective being to observe the biomarker trend over a period (*n* = 13). This meant that most data collected for this review came from secondary outcome measures in the individual papers included.

Biomarkers examining vasomotor tone were commonly examined with renin and adrenomedullin accounting for 10 studies. Renin angiotensin aldosterone system (RAAS) dysregulation has been implicated within vasoplegic states, both with lack of conversion of angiotensin I to angiotensin II and with AT1R receptor down-regulation implicated and elevated levels of circulating renin could indicate this pattern of dysregulation. Both Kullmar et al. [12], and Coulson et al. [3] found that greater renin concentrations were associated with increased vasopressor requirements following cardiac surgery, whilst Montgomery et al. [14] found that increased plasma renin activity was associated with vasoplegia following cardiac surgery. Nguygen et al. [27] investigated renin within the septic cohort and found that increased plasma renin concentration was associated with greater haemodynamic instability. These are consistent with findings from Bellomo et al. [5], who investigated the Angiotensin I/Angiotensin II ratio within the ATHOS-3 population and found that patients with vasodilatory shock had higher baseline levels of angiotensin I compared to healthy volunteers. Dipeptidyl peptidase 3 (DPP3) is an enzyme involved in the degradation of multiple peptides within RAAS, and can result in elevated angiotensin I/angiotensin II ratio and renin levels. Elevated circulatory DPP3 levels have been implicated in the RAAS alterations associated with poor prognosis of circulatory failure patients and could account for why some patients respond poorly to angiotensin. It has the possibility to act as a target for therapeutic intervention [37].

Adrenomedullin is a vasodilatory peptide secreted from the endothelium in response to shear stress, inflammatory insult and trauma, it is also secreted from the adrenal medulla in response to stress. Five studies investigated adrenomedullin in relation to vasoplegia. Three found that elevated levels of adrenomedullin were associated with vasoplegia [6, 28, 38]. Adrenomedullin concentration was negatively correlated with MAP [39] and was predictive of prolonged vasopressor dependence post operatively [16], a high baseline level was also predictive of subsequent development of shock [38]. Both septic patients and those post cardiothoracic surgery were included within these studies, with similar findings, suggesting that the relationship may not be specific to the aetiology of the vasoplegia.

Disappointingly this review did not highlight any studies involving arginine vasopressin (AVP) directly related to vasoplegia. There were two studies covering copeptin as a surrogate marker. Both Pasero et al. [13] and Colson et al. [20] found that higher baseline copeptin values were predictive of post-bypass vasoplegia with the best predictive value for copeptin plasma concentration being >16.9 pmol/L (AUC 0.86, 95% CI 0.73–0.94; OR 1.17, 95% CI 1.04–1.32) and 9.43 pmol/l (with a sensitivity of 90% and a specificity of 77%) respectively. This suggests an activation of the AVP system before surgery that may facilitate depletion of endogenous AVP stores causing post operative vasoplegia.

**Table 1:** Biomarkers being analysed and number of studies investigating these grouped according to mechanism related to vasoplegia

Vasomotor tone	Number of studies
Angiotensin I/angiotensin II	1
Active renin content or plasma renin activity	5
Endothelin + endothelin 1	2
Adrenomedullin	5
Copeptin	2
Calcitonin gene related peptide	1
IDO activity (tryptophan metabolism to kyneyrenine)	1
*Antioxidants*	
Glutathione peroxidase	1
Myeloperoxidase	1
Serum sestrin 2	1
*Related to nitric oxide*	
ADMA	1
l-arginine	1
Adenosine	3
Nitric oxide synthase	2
*Inflammatory mediators/acute phase proteins*	
IL-6	4
IL-10	1
IL-2	1
IL-8	2
TNF	1
FAS/Fas ligand	1
HMGB1	1
OPG	1
Pentraxin 3	1
Heparin binding protein	4
Plasma gelsolin	2
Phospholipase A2	1
*Endothelium/glycocalyx*	
Angiopoietin II	1
Heparan sulfate	1
*Adhesion molecules*	
ICAM-1	2
VCAM-1	1
E-selectin	1
SP-selectin	1
VEGF	1
*Others*	
DPP3	1
Troponin	2
BNP	1
Ischaemia modified albumin	1
VWFBP	1
Vitamin D	1

### Vasoplegia definition

**Fig. 2:**
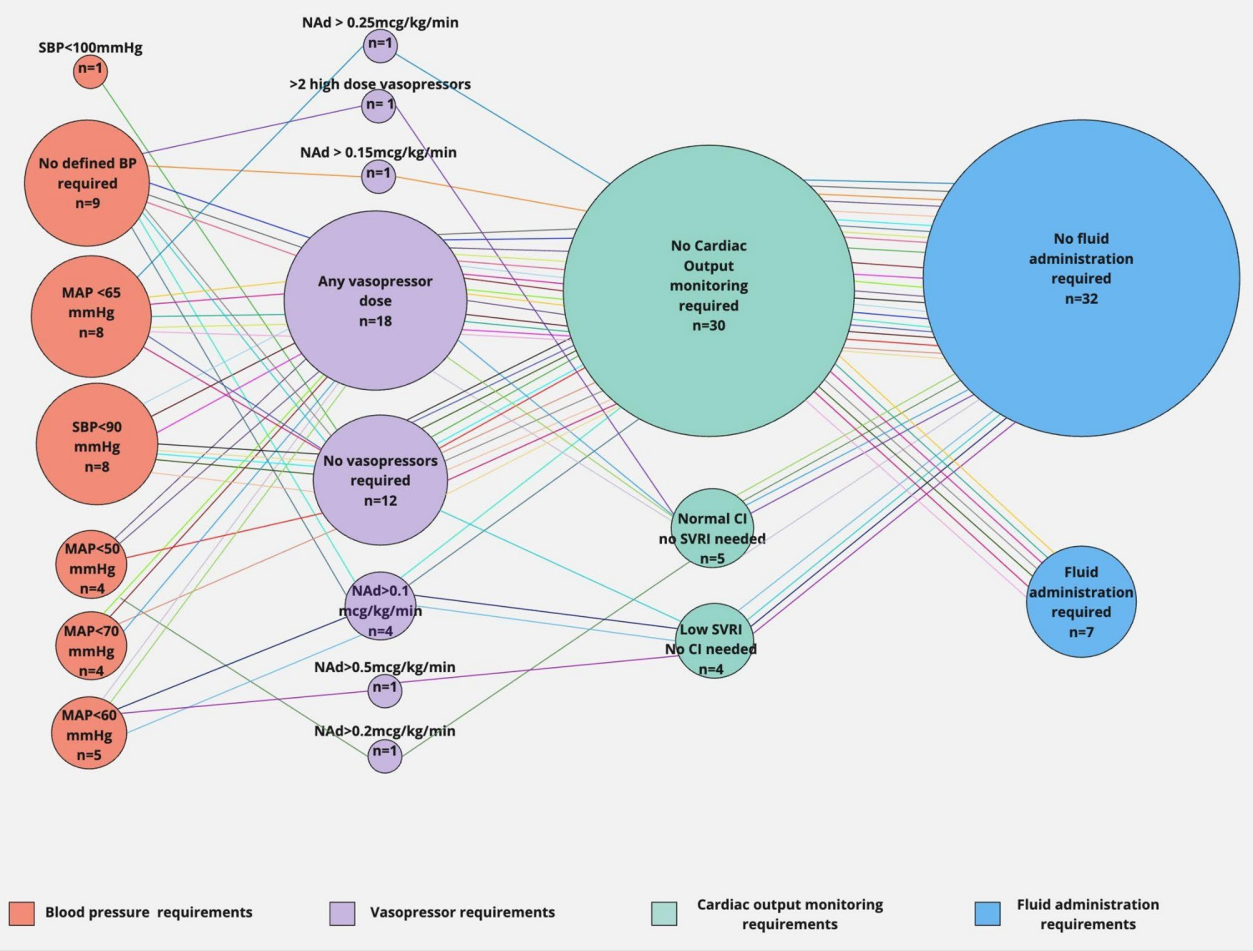
Definitions of vasoplegia across all studies included within the review. This network diagram demonstrates how the different studies defined vasoplegia. Size of the circle is related to the number of times the variable was used. Each linking line has a different colour for each study’s definition. Variables have been divided according to vasopressor requirements, blood pressure requirements, cardiac output monitoring and need for fluid resuscitation. Systolic blood pressure (SBP), mean arterial pressure (MAP), NAd (noradrenaline), cardiac index (CI) systemic vascular resistance (SVRI) This figure has been created using miro.com

There was substantial variation in the definition of vasoplegia that was applied between studies (Fig. 2). Some had strict criteria for the diagnosis of vasoplegia requiring the use of cardiac output monitoring, knowledge of indexed systemic vascular resistance (SVRI) and specific vasopressor doses. By contrast, some used more rudimentary measures to diagnose vasoplegia, such as systolic blood pressure unresponsive to fluid resuscitation. Despite fluid resuscitation commonly being considered a prerequisite, only 7 studies specified this. Four studies, Kullmar et al., Nakamura et al., Rosjo et al., and Beran et al. did not define parameters for diagnosing vasoplegia [12, 24, 29, 40]. It was not specified which noradrenaline salt was used in the administration of noradrenaline in the studies reviewed. Given it is known that there can be significant dosing variations depending upon salt type administrated [41], the preparation used could impact the classification of patients with vasoplegia, particularly when definitions require certain minimum doses to be required to allow the diagnosis.

## Discussion

### Summary of main findings

This systematic review identified 43 studies investigating 39 different biomarkers across various vasoplegic states, predominantly in sepsis and post-cardiac surgery populations. The most extensively studied biomarkers were renin and adrenomedullin (five studies each), followed by heparin binding protein and IL-6 (four studies each). Our review revealed substantial heterogeneity in both the definition of vasoplegia used across studies and the methodological approaches employed, which precluded meta-analysis despite multiple studies examining the same biomarkers.

Several biomarkers demonstrated potential clinical utility. Renin, a key component of the renin-angiotensin-aldosterone system, showed consistent associations with vasoplegic states across multiple studies, with elevated levels corresponding to increased vasopressor requirements in both cardiac surgery and septic populations. Similarly, adrenomedullin emerged as a promising biomarker with predictive capabilities for shock development and vasopressor dependence, showing relevance across different vasoplegic aetiologies.

### Strengths and limitations

This systematic review has several notable strengths. First, it represents the first comprehensive examination of biomarkers across the entire spectrum of vasoplegic states in critical care, allowing for comparison of biomarker profiles across different aetiologies. Second, our broad inclusion criteria and extensive search strategy, including manual reference searching, ensured comprehensive capture of relevant literature despite the challenging terminology around vasoplegia. Third, the systematic documentation of vasoplegia definitions across studies provides valuable insight into the variability of diagnostic approaches currently employed in research and clinical practice. Fourth, the categorization of biomarkers by physiological mechanism offers a framework for understanding the complex pathophysiology underlying vasoplegia. Finally, the rigorous methodology employed, including independent review by multiple authors and formal risk of bias assessment using QUADAS-2, enhances the reliability of our findings and transparency regarding limitations of the included evidence.

There are several limitations within this systematic review. Primarily, creating a search strategy was challenging due to the varied terms used to describe vasoplegia and the lack of a MeSH term for this prior to 2010. As broad search terms were used, generating large numbers of papers initially, it remains possible that we missed some manuscripts. To mitigate this, we manually searched through references from papers including relevant review articles. This mitigation only yielded a further nine full texts to review, with only three of these being included within the final review.

We were unfortunately unable to perform a meta-analysis despite six biomarkers having three or more studies investigating them. This was primarily due to differences in assay type and different outcome measures between the studies. Another barrier to meta-analysis was that the reported comparisons varied. For example, some studies compared concentrations between two groups (i.e., vasoplegia versus control) whilst others reported trends of concentrations within a group (all vasoplegic), or correlated plasma concentrations to the degree of vasoplegia demonstrated.

The review highlighted significant methodological challenges in biomarker research for vasoplegia, including variations in measurement techniques, timing of sample collection, and inconsistent reporting of outcomes. Most notably, we identified a critical lack of standardization in the definition of vasoplegia itself, with studies using diverse criteria ranging from comprehensive hemodynamic parameters with cardiac output monitoring to simple blood pressure measurements with vasopressor requirements. Given these highly pragmatic definitions, or in some cases no clearly defined definition, it is possible that some patients may have exhibited hypotension due to aetiologies other than a reduction in SVR and vasodilation, making comparisons between different phenotypes of shock potentially redundant. Additionally, the broad range in definitions means that the severity of physiological disturbance included within this review may be so dissimilar that meaningful comparison between different cohorts or even within the same cohort (e.g., sepsis) could be limited.

### Comparison with existing literature

To our knowledge this review represents the only systematic review on the topic of biomarkers relating to all aetiologies of vasoplegia as well as the definition of vasoplegia. There have been many narrative reviews on the topic, however these have all be confined to looking at characteristics of individual phenotypes (e.g. sepsis or post cardiac bypass). This review has also highlighted a lack of literature looking at vasoplegia and biomarker utilisation within populations other than septic shock and cardiothoracic surgery. We know that this is a phenomenon that happens across a variety of different aetiologies, however these have not been represented within this review or within the current literature.

### Interpretation of results

Both renin and adrenomedullin have shown predictive capabilities, with Kullmar et al. showing that a large change in renin between pre and post operative levels were predictive of vasoplegia development following cardiac surgery [12]. Caironi et al. found that adrenomedullin was predictive of shock development [38] in septic patients presenting to the emergency department. In terms of underlying mechanisms; studies involving both renin and adrenomedullin have included septic and post cardiac surgery patients, for example with adrenomedullin, both Marino et al. and Van Lier et al. found that adrenomedullin was correlated with degree of hypotension in both septic and cardiac surgery patients, suggesting that adrenomedullin may be implicated in both aetiologies [16, 39].

### Implications for clinical practice

This systematic review has several implications for clinical practice, including highlighting the potential that emerging biomarkers may have for both the prediction of vasoplegic shock following a known insult and allow risk stratification for those showing early signs of shock. This could aid early decision making in these patients with regard to potential therapeutic approaches. With the increasing advent of point of care tests, along with assays becoming more accessible, this may become less of a barrier in the future.

### Implications for research

This review has highlighted substantial variation in how the terms “vasoplegia” and “vasoplegic shock” are used within the literature. Establishing consensus definitions would facilitate evidence synthesis from disparate studies and provide a foundation for future research. However, achieving this consensus presents significant challenges. A fundamental tension exists between developing highly specific definitions versus pragmatic ones. A comprehensive definition incorporating blood pressure criteria, hemodynamic parameters, minimum intravenous fluid resuscitation volumes, and vasopressor requirements would be thorough but not always achievable outside rigorous trial protocols. Conversely, a more pragmatic definition that omits these criteria risks including patients with hypotension of different aetiologies (such as hypovolaemia), thereby compromising trial validity.

The two most commonly studied variants of vasoplegia are septic shock and cardiac surgery-associated vasoplegia, with several attempts at developing specific definitions for these conditions [5, 6, 13, 18, 19]. A less studied but globally common variant is vasopressor infusion following non-cardiac surgery, recently described in detail [42].

Since current therapies for vasodilatory shock are not aetiology-specific, a common definition would be particularly valuable. Future clinical advances may refine vasopressor selection based on point-of-care biomarker measurements [12]. To explore the potential advantages of biomarker stratification, it may be beneficial to recruit patients across all aetiologies of vasodilatory shock. Through comprehensive biomarker analysis, researchers could identify putative mechanisms shared across clinical phenotypes, thereby defining distinct endotypes that transcend traditional diagnostic categories.

## Conclusion

There remain many unanswered questions surrounding the pathophysiology of vasoplegia, to date studies of biomarkers of vasoplegia in humans have been too heterogenous to allow meta-analysis. In addition to biomarkers’ potential to diagnose, predict and prognosticate, they can also offer mechanistic insights into pathophysiology. Three fundamental improvements are required to progress this field: (i) a pragmatic consensus definition of vasoplegia, (ii) consensus on the most appropriate times to sample and standardised processing and analysis (iii) use of standardised definitions for outcomes. Further studies are required that investigate biomarkers for their ability to predict the development of vasoplegia as well as the severity of the condition. This will in turn aid the development of improved therapies for vasoplegia.

## Supplementary Information

Supplementary Material 1.

## Data Availability

The datasets used and/or analysed during the current study are available from the corresponding author on reasonable request.

## Abbreviations

ADMA: Asymmetric dimethylarginine
AT1R: Angiotensin 1 receptor
AVP: Arginine vasopressin
BNP: Brain natriuretic peptide
DAMPs: Damage associated molecular patterns
DPP3: Dipeptidyl peptidase 3
ECMO: Extra corporeal membrane oxygenation
HMGB1: High mobility group box 1
ICAM-1: Intra cellular adhesion molecule 1
ICU: Intensive care unit
LVAD: Left ventricular assist device
OPGO: Steoprotegerin
PAMPS: Pathogen associated molecular patterns
QUADAS-2: Quality assessment of studies of diagnostic accuracy
TNF: Tumour necrosis factor
VCAM-1: Vascular cell adhesion molecule 1
VEGF: Vascular endothelial growth factor
VWPBF: Von Willebrand protein binding factor
