# Supplementary material for: Circulating biomarkers of vasoplegia: a systematic review
Source: Ann Intensive Care. 2025 Sep 30;15:150. doi: 10.1186/s13613-025-01564-7 (PMC12480150; doi:10.1186/s13613-025-01564-7)
Supplement: Supplementary file 1 — Supplementary Material 1. [file 13613_2025_1564_MOESM1_ESM.docx]

# Appendix 1: PRISMA flow diagram

PRISMA 2020 flow diagram of study search and exclusions


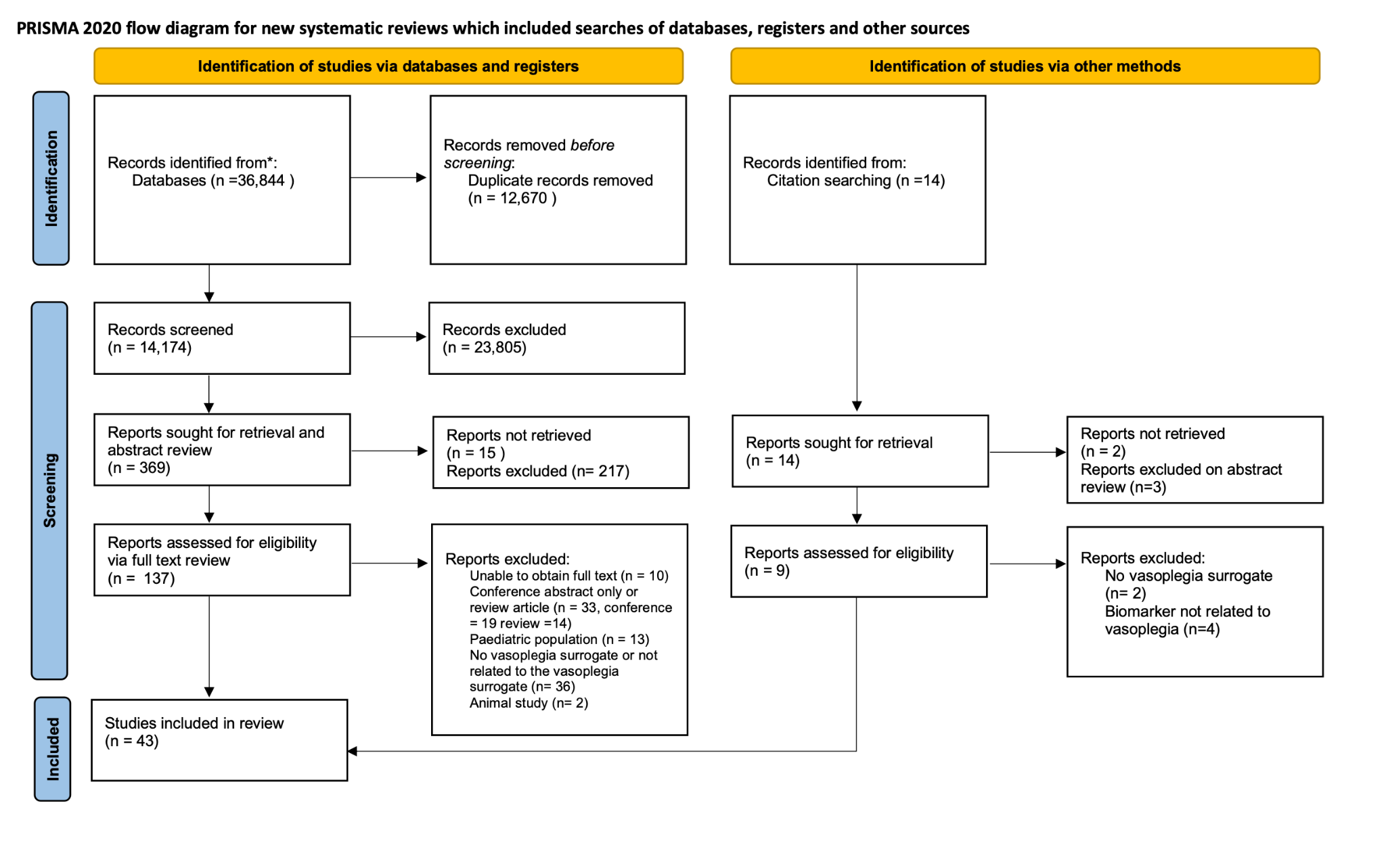


# Appendix 2: Additional Figures

Figure 1: Number of studies grouped by design

Figure 2: Number of studies grouped by diagnosis subtype


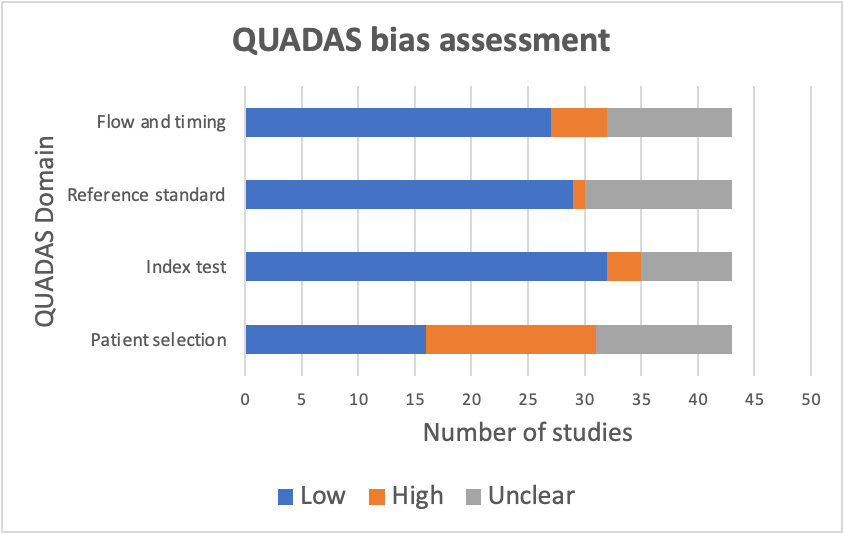


Figure 3: QUADAS bias assessment, blue = low risk, orange = high risk, grey = unclear

# Appendix 3: Summary tables of all studies included

Divided by mechanism

| **Author,**  **Year** | **Biomarker** | **Population** | **Comparator/ control** | **Vasoplegia definition** | **Population size, % incidence of vasoplegia** | **Study primary outcome measure**  **Vasoplegia surrogates related to biomarker analysis if not primary outcome measure of study** | **Results** |
| --- | --- | --- | --- | --- | --- | --- | --- |
| **VASOMOTOR TONE** | | | | | | | |
| Renin | | | | | | | |
| Montgomery et al, 2023 [36] | Plasma Renin Activity | Post cardiac surgery | Vasoplegic vs non vasoplegic post operatively | Noradrenaline equivalent dose of >0.15 mcg/kg/min at any point on post operative day 1(POD1) | 100, 13% | Incidence of vasoplegia compared to plasma renin activity | Median plasma renin activity at baseline 5.7 vs 1.3 ng/kg/hr p=0.01 associated with vasoplegia |
| Jeyaraju et al, 2022 [27] | Plasma renin concentration | Vasoplegia of any aetiology | No control | Vasopressors for >6h to maintain MAP >65mmHg | 53, 100% | Primary outcome:  change in plasma renin con-  centration over time would be superior to the change in lactate concentration for  predicting in-hospital mortality in hypotensive patients on vasopressors. | Plasma renin  kinetics may be superior to lactate kinetics in predicting  mortality of hypotensive, critically ill patients. |
| Kullmar et al, 2021 [34] | Plasma renin concentration kinetics  Change in renin pre operatively to post operatively stratified to high or low delta renin. | Post cardiac surgery | Vasoplegic vs non vasoplegic post operatively | Not defined within protocol | 197, unknown vasoplegia incidence | Primary outcome: incidence of AKI  Delta renin related to vasoplegia incidence, vasopressor dose and severity of hypotension | High delta renin associated with higher vasopressor dose (P=0.002), lower MAP (p=0.016) not associated with vasoplegia incidence (p=0.15) |
| Nguygen et al, 2019 [29] | Plasma renin concentration | Septic shock | No control | Noradrenaline requirements >0.25mcg/kg/min | 40, 100% | Primary outcome:AKI incidence, prediction of persistent AKI and adverse renal outcomes  Correlation of renin concentration with vasopressor dose and MAP | Correlation between renin concentration and vasopressor dose r=0.36, p=0.02, correlation between renin concentration and MAP r=-0.55, p<0.01 |
| Coulson et al, 2011 [15] | Plasma renin concentration | Cardiac surgery | No control | Noradrenaline or Angiotensin II infusions to maintain MAP 70-80mmHg | 60, 100% | Primary outcome =  Noradrenaline and Angiotensin II requirements vs plasma renin concentration | Higher baseline plasma renin conc associated with greater noradrenaline requirements (p=0.003), not angiotensin II requirements (p=0.54) |
| Adrenomedullin | | | | | | | |
| Hillinger et al, 2022 [18] | Plasma mid regional pro- adrenomedullin concentration | Post cardiac surgery | Cardiac surgery without vasoplegia | MAP<60mmHg + SVRI <1200 + Noradrenaline >0.1mcg/kg/min for 24h | 42, 76% | Primary outcome = to explore the post operative course of adrenomedullin  Adrenomedullin concentrations and those with vasoplegia vs those without.  Vasopressor dose required and adrenomedullin concentration | Higher ADM for anyone requiring Noradrenaline at all time points, p<0.001. No significant association between adrenomedullin levels and maximum vasopressor dose required |
| Van lier et al, 2022 [39] | Bio- adrenomedullin (Bio-ADM) | Post cardiac surgery | Cardiac surgery without vasoplegia | MAP <70mmHg not responsive to fluid administration with CI >2.5L/min/m^2 | 208, 15.6% | Primary outcome: to investigate the temporal profile of bio-ADM  Is bio-ADM able to predict prolonged vasopressor dependency | Day 2 bio-ADM was the best predictor of those with prolonged vasopressor requirements AUROC 0.82, p<0.001 |
| Caironi et al, 2017 [26] | Bio- adrenomedullin (Bio-ADM) | Sepsis | Septic not shocked | Score of 3 or 4 in the cardiovascular component of the SOFA score | 956, 39% | Primary outcome: association of bio-ADM and its time dependent variation with fluid therapy, vasopressor administration, organ failure and mortality. | Day 1 Bio-ADM higher in patients with septic shock p<0.0001, High day 1 Bio-ADM predictive of subsequent haemodynamic compromise |
| Marino et al, 2014 [38] | Adrenomedullin concentration | Sepsis + septic shock | Septic not shocked | Use of vasopressors to maintain MAP>65mmHg | 101, 17.8% | Primary outcome: assess use of adrenomedullin levels and correlation with outcomes.  Adrenomedullin related to those with shock and vasopressor requirements | Plasma ADM levels negatively correlated with mean arterial pressure (r = −0.39; P <0.0001), and median ADM strongly discriminated those requiring vasopressor therapy from the others 129 pg/ml vs 48pg/ml p<0.0001 |
| Nishio et al, 1997 [37] | Adrenomedullin concentration (ADM) | Sepsis + Septic shock | Healthy volunteers | SBP< 90mmHg not responsive to fluid administration | 25,  12 with septic shock | Primary outcome:  Trends of ADM in patients with septic shock and investigation associations of ADM concentrations with relaxation of vascular tone | Mean ADM concentrations higher in shocked cohort 226.1 fmol/ml vs 5.05 fmol/ml p<0.001  Mean ADM concentration correlated with decreasing diastolic pressure (r=-0.39 p<0.05). Non significant relation of ADM to SVRI and CO changes. |
| Endothelin 1 | | | | | | | |
| Hillinger et al, 2022 [18] | Plasma CT-pro ET1 (c terminal pro endothelial 1) concentration | Cardiac surgery | Cardiac surgery without vasoplegia | MAP<60mmHG + SVRI <1200 + Noradrenaline>0.mcg/kg/min for 24hours | 42, 76% | Primary outcome: to explore the post operative course of CT-pro ET1  Ct-pro ET1 concentrations and those with vasoplegia vs those without.  Vasopressor dose required and CT-pro ET1 concentration | Higher Ct-pro ET1 for anyone on vasopressors p<0.001 |
| Avontuur et al, 1999 [16] | Endothelin 1 plasma (ET-1) concentration | Septic shock | Healthy volunteers | SBP< 90mmHg requiring vasopressors | 22,  11 healthy controls recruited | Primary outcome: whether ET-1 contributed to the vasopressor action of the NO synthase inhibitor NG-nitro-L-arginine methyl  ester in patients with severe septic shock.  Comparison of ET-1 levels between septic shock and healthy volunteers | Compared with healthy volunteers, plasma levels of ET-1, nitrate/nitrate, and cortisol were significantly increased (p<0.05) |
| Copeptin | | | | | | | |
| Pasero et al, 2020 [57] [(26)](https://www.zotero.org/google-docs/?vuVrLE) | Copeptin concentration | Cardiac surgery | Cardiac surgery without vasoplegia | MAP <60mmHg, SVRI <1200, norad >0.1 for 12h | 55, 16.3% | Primary outcome: trend of biomarker postoperatively  Ability to predict development of vasoplegia | Pre-operative copeptin >16.9pmol/l predicted vasoplegia AUROC 0.86 |
| Colson et al, 2011 [58] | Plasma copeptin concentrations | Cardiac surgery | Cardiac surgery without vasoplegia | < 60 mmHg with cardiac index ≥ 2.2 l/min/m^2, given NAd | 64, 15.6% | Primary outcome: association of copeptin concentrations and incidence of vasoplegia, the use of copeptin to predict post operative vasoplegia | Vasopleigc patients had significantly higher copeptin plasma concentration before  cardiopulmonary bypass (P < 0.001).  The best predictive value for preoperative  copeptin plasma concentration was 9.43 pmol/l with a sensitivity of 90% and a specificity of 77%. |
| Angiotensin I/II | | | | | | | |
| Bellomo et al, 2020 [17] | Plasma Angiotensin I (ANG I)and Angiotensin II (ANG II) concentration and their ratio | Vasoplegic shock | Healthy volunteers | Noradrenaline >0.2mcg/kg/min for MAP 55-70mmHg or ScvO2>70, CI>2.3L/min/m^2 | 321, + 24 healthy volunteers  (ATHOS 3 population) | Primary outcome: measure Angiotensin I/II ratios and assess outcome associations of the population  Comparison of biomarker levels between shocked group and healthy population | Patients with vasodilatory shock had higher median baseline ANG I levels, 253ng/mL, vs 42pg/mL p<0.001.  Median ANG II levels were similar 84pg/mL vs 97pg’mL p=0.99 between the two groups. |
| Calcitonin gene related peptide | | | | | | | |
| Arnalich et al, 1996 [33] | Calcitonin gene related peptide (CGRP) concentration | Sepsis | Septic not shocked | SBP<90mmHg despite fluid resuscitation | 31, 42% | Primary outcome: evidence to support the involvement of CGRP in hemodynamic changes seen in septic shock | CGRP concentration significantly higher at all time points in the septic shock cohort |
| Tryptophan to Kynurenine metabolism (Indoleamine 2,3-dioxygenase activity activity) | | | | | | | |
| Changsirivathanathamrong et al, 2011 [31] | Indoleamine 2,3-dioxygenase (IDO) activity | Septic shock | Healthy volunteers | MAP<65 mmHg requiring noradrenaline following fluid resuscitation | 16  9 healthy volunteers | Primary outcome: assess relationship of IDO activity and degree of hypotension | IDO activity in- creased up to ninefold in patients with septic shock and was significantly higher than in the two control groups (p < .01). IDO activity was strongly correlated with inotrope requirements (p < .001) |
| **ADHESION MOLECULES** | | | | | | | |
| ICAM-1 | | | | | | | |
| Schuetz et al, 2011 [19] | ICAM-1 plasma levels | Septic shock | Non vasoplegic shock:  Cardiogenic & Haemorrhagic | SBP < 100 mmHg | 161, 42.9% | Primary outcome: biomarker comparison between different aetiologies of shock | Multivariate logistic regression analysis of ICAM-1 did not reach statistical significance (p=0.17) in septic shock vs control group |
| Sessler et al, 1995 [32] | Plasma circulating ICAM-1 levels (cICAM-1) | Sepsis + SIRS in ICU | ICU patients without sepsis  Septic not shocked  Non vasoplegic shock  Healthy volunteers | SBP <90mmHg, need for noradrenaline infusion | 66, 24% | Primary outcome: trends of cICAM-1 in septic adults, relationship between cICAM-1 and disease severity  Association between cICAM-1 and development of shock/vasoplegia | Day 1 cICAM-1 lev-  els were higher (p = 0.017, ANOVA) in 16 patients with septic shock than in seven with severe sepsis and two with sepsis but without hypotension or hypoperfusion. There was a positive correlation (r =0.50, P = 0.009) between Day-1 cICAM-1 measurements and severity of shock as determined by the presence of  hypotension and vasopressor use. |
| VCAM-1 | | | | | | | |
| Schetuz et al, 2011 [19] | VCAM-1 levels | Septic shock | Non vasoplegic shock:  Cardiogenic& Haemorrhagic | SBP < 100 mmHg | 161, 42.9% | Primary outcome: biomarker comparison between different aetiologies of shock | Multivariate logistic regression analysis of VCAM-1 did not reach statistical significance (p=0.1)in septic shock vs control |
| Selectins | | | | | | | |
| Kortekaas et al, 2013 [59] | SP Selectin levels | Cardiac surgery | Cardiac surgery without vasoplegia | PCSV was defined as mean arterial blood pressure < 60 mmHg with cardiac index ≥ 2.2 l/min/m^2, and was treated with norepinephrine to restore mean blood pressure > 60 mmHg | 40, 37.5% | Primary outcome: could pre-existing endothelial activation (evidenced by biomarkers) predict post operative vasoplegia | Pre-operative levels with ROC curve analysis showed cut-off value 64.4 ng/ml with accuracy indicating Vasoplegia (r=0.45, P=0.004)  120% increase in sp-selectin levels pre to post operatively in vasoplegic patients p=0.03  Baseline levels of SP selectin higher in vasoplegic 76.0ng/ml vs 54.0ng/ml p=0.006 |
| Schuetz et al, 2011 [19] | E-selectin levels | Septic shock | Non vasoplegic shock:  Cardiogenic& Haemorrhagic | SBP < 100 mmHg | 161, 42.9% | Primary outcome: biomarker comparison between different aetiologies of shock | Multivariate logistic regression analysis showed significant association of E-Selectin (OR 3.7, 95% confidence interval: 1.7-7.8, p=0.001) with sepsis aeitology |
| VEGF | | | | | | | |
| Schuetz et al, 2011 [19] | VEGF | Septic shock | Non vasoplegic shock:  Cardiogenic& Haemorrhagic | SBP < 100 mmHg | 161, 42.9% | Primary outcome: biomarker comparison between different aetiologies of shock | VEGF did not reach statistical significance (p=0.06) |
| **RELATED TO NITRIC OXIDE** | | | | | | | |
| Adenosine | | | | | | | |
| Nee et al, 2013 [46] | Adenosine plasma concentration (APC) | Cardiac surgery | Cardiac surgery without vasoplegia | MAP <50mmHg requiring, vasopressors | 86, 19.8% | Primary outcome: APL change with vasoplegia following cardiac surgery  APC in vasoplegic vs non complicated group | APC significantly higher in the vasoplegic cohort at all time points measured (p<0.05) |
| Kerbaul et al, 2008 [30] | Adenosine A2A receptor expression  Adenosine plasma levels (APL) | Cardiac surgery | Cardiac surgery without vasoplegia  Healthy volunteers | MAP <50mmHg, requiring vasopressors | 44, 22.7%  10 healthy as a separate subgroup | Primary outcome: to evaluate APLs and A2A Receptor expression in patients with or without vasoplegic shock following cardiac surgery and in healthy controls | APL significantly higher in vasoplegia vs uncomplicated,and again in uncomplicated vs healthy,  MAP inversely correlated with APL, R -0.58 p<0.001.  Receptor expression higher in vasoplegic vs uncomplicated and healthy (no diff between uncomplicated and healthy) |
| Kerbaul et al, 2006 [45] | Adenosine plasma level (APL) | Cardiac surgery | Cardiac surgery without vasoplegia | MAP <50mmHg, requiring vasopressors | 35, 20% | Primary outcome:  APLs were correlated to operative and postoperative clinical courses.  Association of APL with vasoplegia vs control | APLs were significantly  higher in vasoplegic cohort (1.6 mol·L1 [0.2–2.6] vs. 0.4 mol·L1 [0.1–1.0]) p<0.05 |
| L-arginine/arginine/ADMA | | | | | | | |
| Nakamura et al, 2009 [35] | Serum asymmetric dimethylarginine (ADMA) levels | Septic shock | Healthy volunteers | Not defined | 30, 15 septic shock | Primary outcome: correlation of ADMA levels in patients with septic shock compared to healthy individuals | ADMA levels significantly higher in septic shock patients compared to healthy individuals, p<0.0001 |
| Villalpando et al, 2006 [52] | In vivo arginine production (flux) | Septic shock | Healthy volunteers | Noradrenaline requirements >0.1mcg/kg/min | 16, 37.5% | Primary outcome: measure in vivo arginine production and the  intravascular NO synthesis rate in hypotensive septic patients.  Plasma arginine concentrations between vasoplegic and healthy groups | Slower arginine flux (99 vs 50 umol/kg/h,  p=0.01)  Lower plasma arginine concentrations (75 vs 40 umol/L p= 0.01), |
| Nitric Oxide and Nitric Oxide Synthase | | | | | | | |
| Huttunen et al, 2009 [51] | NOS genotyping | Sepsis | Septic not shocked | MAP <70 mmHg requiring vasopressors | 147, 21% | Primary outcome: determine the role of the G894T polymorphism of the eNOS gene on the clinical course of patients with bacteraemia  Association of the genotype with hypotension and septic shock | T allele carriage is a significant risk factor for hypotension, (OR, 10.8; 95% CI, 2.0-57.7; P = 0.005),  & associated with lower MAP.  More hypotensive patients among the T-allele carriers compared with noncarriers P = 0.015 |
| Villalpando et al, 2006 [52] | Nitric Oxide (NOx) | Septic shock | Healthy volunteers | Noradrenaline requirements >0.1mcg/kg/min | 16, 37.5% | Primary objective: measure in vivo  intravascular NOx synthesis rate in hypotensive septic patients. | The septic shock group had significantly  higher plasma NOx concentrations p<0.01 |
| **ANTIOXIDANTS** | | | | | | | |
| Glutathione Peroxidase | | | | | | | |
| Semedi et al, 2023 [22] | Glutathione peroxidase (GPx) | Vasodilatory shock | No control | “Shock” after 1000 ml fluids and requiring NAd >0.1mcg/kg/min | 34, 100% | Role of antioxidants and its relation to disease severity  Relation between vasopressor index score (VIS) and biomarker levels  Relation between MAP and biomarker levels | Non significant correlation between GPx levels and MAP (r=0.15 p=0.395), and non significant correlation between GPx levels and VIS (r=-0.01 p0.978) |
| Sestrin 2 | | | | | | | |
| Huang et al, 2022 [55] | Serum sestrin 2 | Sepsis | Healthy volunteers | MAP <65mmHg requiring vasopressors after fluid resuscitation | 227, 82.8% | Primary outcome: differences in serum sestrin 2 between septic patients and healthy cohort  Association between SS2 and noradrenaline requirements, incidence of vasoplegia | Sestrin2 levels showed a significant difference between septic shock and healthy controls  (12.4 ng/ml vs 5.8 ng/ml, p < 0:01).  Non significant negative association between sestrin2 levels and norepinephrine dose |
| Myeloperoxidase | | | | | | | |
| Sef et al, 2023 [24] | Myeloperoxidase (MPO) concentrations | Lung transplant | Lung transplant not shocked | MAP< 60mmHg, SVR <800dyn/s/cm, CI>2.5l/min/m^2 | 279, 41.6% | Primary outcome: describe the incidence of vasoplegia in lung transplant recipients.  Biomarker analysis and relation to vasoplegia as sub analysis | Nonsignificant higher  MPO in the vasoplegic group at baseline  and up to 24 h postoperatively. |
| **ENDOTHELIUM/GLYCOCALYX** | | | | | | | |
| Abou-Arab et al, 2020 [60] | Heparan sulfate + syndecan 1 | Cardiac surgery | Cardiac surgery without vasoplegia | MAP <65 despite preload optimisation needing Noradrenaline for >4 hours | 46, 37% | Primary outcome: comparison of biomarker levels in patients with and without vasoplegia post operatively | Syndecan 1 levels lower at all timepoints in vasoplegic group.  Baseline plasma syndecan 1 predicted development of vasoplegia with AUROC 0.7 p=0.045 w/ 95% CI (lower levels associated with vasoplegia)  Heparan sulfate levels not modified by cardiac surgery |
| Kortekaas et al, 2013 [59] | Angiopoeitin II | Cardiac surgery | Cardiac surgery without vasoplegia | MAP < 60 mmHg with cardiac index ≥ 2.2 l/min/m^2, requiring Noradrenaline for MAP > 60 mmHg | 40, 37.5% | Primary outcome: could pre-existing endothelial activation (evidenced by biomarkers) predict post operative vasoplegia | No significant difference in angiopoietin II levels between vasoplegic and non vasoplegic, 2.5ng/ml vs 2.4ng/ml p=0.57 |
| **INFLAMMATORY MEDIATORS/ ACUTE PHASE PROTEINS** | | | | | | | |
| Interleukins and inflammatory mediators | | | | | | | |
| Tsay et al, 2016 [23] | IL-6 | Sepsis | Septic not shocked | SPB <90mmHg | 85, unknown | Primary outcome: evaluate changes in IL-6 within the septic population  Comparison between the septic shock and septic not shocked groups | No association with septic shock and IL-6 levels  Non significant association with SBP and IL-6 |
| Kortekaas et al, 2013 [59] | IL-8, OPG, VWFF | Cardiac surgery | Cardiac surgery without vasoplegia | MAP < 60 mmHg with cardiac index ≥ 2.2 l/min/m^2, requiring Noradrenaline for MAP > 60 mmHg | 40, 37.5% | Primary outcome: could pre-existing endothelial activation (evidenced by biomarkers) predict post operative vasoplegia | Baseline levels of VWF propeptide higher in those who developed vasoplegia, 25.4 nM vs 8.4nM p<0.001  Non significant lower baseline OPG in those with vasoplegia 26.3 pg/ml vs 38.9 pg/ml p=0.09  Non significant difference in IL-8 between vasoplegic and non vasoplegic, 8.3pg/ml vs 8.4 pg/ml p=0.45 |
| Beran et al, 2010 [21] | Il-6, IL-8, IL-10 | Sepsis | No control (case series) | Not defined | 3, 100% | Case series | IL-6, IL-8, IL-10 levels all highest on day 1 of shock, with decreasing levels over consecutive days  (no statistics applied in paper) |
| Nakamura et al, 2009 [35] | IL-6 + HMGB1 | Sepsis | Healthy volunteers | Not defined | 30  15 healthy | Primary outcome: correlation of IL-6 levels in patients with septic shock compared to healthy individuals | Serum levels of IL-6 significantly higher in septic cohort p < 0.0001  Serum levels of HMGB1 significantly higher in septic cohort p < 0.0001 |
| Pinsky et al, 1993 [43] | IL-6, IL-2, TNF | Sepsis | Other shock, not clearly defined | MAP <50mmHg requiring vasopressors >12hours | 53, 100% | Primary outcome: difference between levels related type of shock | TNF and IL-6 higher in patients with septic shock vs non septic shock P<0.05.  No significant difference with IL-2 levels between the two groups |
| Huttunen et al. 2012 [50] | Fas Ligand/ Soluble Fas (sFas) | Sepsis | Septic not shocked | MAP<70mmHg | 132, 39% | Primary outcome: prognostic value of apoptosis markers  Relation to shocked vs non shocked | Vasoplegia vs non vasoplegia: Max sFas 10939 vs 9017 p= 0.002, sFas/FASL ratio 374 v 293 p0.024 |
| Heparin Binding Protein (HPB) | | | | | | | |
| Sef et al, 2023 [24] | Heparin binding protein | Lung transplant | Lung transplant not shocked | MAP< 60mmHg, SVR <800dyn/s/cm , CI>2.5l/min/m^2 | 279, 41.6% | Primary outcome: describe the incidence of vasoplegia in lung transplant recipients.  Biomarker analysis and relation to vasoplegia as sub analysis | The postoperative levels of  Heparin binding protein were similar between the two groups. |
| Tverring et al, 2020 [28] | Heparin binding protein | Sepsis | No control | MAP<65 after fluid resuscitation requiring vasopressors | 24, 100% | Primary outcome: explore kinetics of HPB and during septic shock and investigate an  association between repeated measures of HBP concentration and cardiovascular  organ dysfunction severity.  Association of HPB and MAP, SVRI and noradrenaline dose | Every 100 ng/mL increase in HBP corresponded to a 30% increase in  NA dose in a crude model (95% CI 3 to 60%, p = 0.03,  HBP was significantly associated to MAP (95% CI − 0.004 to − 0.3, p = 0.01)  Every 1 ng/mL increase in HBP was associated with 1 dyne s cm−5 m−2 decrease in SVRI in a model adjusting for time, CI and NA dose (95% CI − 0.36 to − 1.62, p = 0.002,) |
| Beran et al 2010 [21] | Heparin binding protein | Sepsis | No control (case series) | Not defined | 3, 100% | Case series | HPB levels all highest on day 1 of shock, with decreasing levels over consecutive days  (no statistics applied in paper) |
| Linder et al, 2009 [44] | Heparin binding protein | Sepsis | Septic not shocked | SBP<90 mmHg requiring vasopressors after fluid resuscitation | 233, 11.1% | Primary outcome: whether plasma levels of HBP could be used as an early diagnostic marker  for septic shock | A plasma HBP level 15 ng/mL was a better indicator of severe sepsis (with or without septic shock) (sensitivity: 87.1%; specificity: 95.1%; PPV: 88.4%; NPV: 94.5%).  HBP elevated in 90% of those who went on to develop septic shock prior to shock symptoms |
| Plasma Gelsolin | | | | | | | |
| El-Magharaby, 2021 [56] | Plasma gelsolin concentration | Sepsis | Septic not shocked  Non vasoplegic shock | Hypotension despite adequate fluid resuscitation | 80, 65% | Primary outcome: investigate the clinical value of plasma gelsolin concentration for the diagnosis of  Sepsis and investigate the relationship between plasma gelsolin concentration and the severity of organ dysfunction  Association between gelsolin concentration and septic shock | Mean pGSN levels higher in septic shock than septic not shocked patients,  Septic patients showed significantly decreased 1st-day GSN levels (170.9 ± 74.3 mg/l) compared to non-  septic critically ill patients (225.9 ± 84.5 mg/l, p < 0.05). |
| Abou-Arab et al, 2019 [60] | Plasma gelsolin | Cardiac surgery | Cardiac surgery without vasoplegia | MAP <65 despite preload optimisation needing noradrenaline for >4hours | 46, 37% | Primary outcome: comparison of biomarker levels in patient with and without vasoplegia post operatively | No difference in gelsolin levels between cohorts |
| Pentraxin 3 | | | | | | | |
| Uusitalo-Seppälä et al, 2013 [53] | Pentraxin 3 plasma concentration (PTX3) | Sepsis | Septic not shocked | SPB<90mmHg, non responsive to 500ml fluid administration | 537, 9% | Primary outcome: evaluate usefulness of PTX3 in risk stratification and prediction of development of septic shock  Association of PTX3 with septic shock | median PTX3 concentration was higher in severe sepsis patients compared to others (16.7 vs. 4.9 ng/ml,  p=0.001)  A high PTX3 level  predicted hypotension (p=0.001).  PTX3 at a cut-off level for 14.1 ng/ml (optimal cut-off value for septic shock) showed 63% sensitivity and 80% specificity.  AUCROC in the prediction of septic shock was 0.73  (95% CI 0.66–0.81, p=0.001) |
| Phospholipase A2 | | | | | | | |
| Vadas et al, 1984 [61] | Phospholipase A2 (PLA2) levels and activity | Septic shock | Healthy volunteers, hypotensive not septic | SBP<90mmHg refractory to volume replacement | 34  21 healthy volunteers  25 hypotensive not septic | Primary outcome: trend of phospholipase A2 levels in septic shock  Differences between phospholipase A2 levels and activity in septic and healthy cohorts | PLA2 activity elevated in septic shock compared to healthy controls  p <0.0001. No difference in PLA2 activity between septic shock and hypotensive non septic patients |
| **OTHER** | | | | | | | |
| Pasero, 2021 [57] | NT- proBNP | Cardiac surgery | Cardiac surgery without vasoplegia | MAP <60mmHg, SVRI <12002.5l/min/m^2  , norad >0.1mcg/kg/min for 12hours | 55, 16.3% | Primary outcome: trend of biomarker postoperatively  Ability to predict development of vasoplegia | Median NT-proBNP was higher among vasoplegic group at T0(1435 pg/mLvs. 365.5 pg/mL  p=0.006), and at T1 (2,053 pg/mL vs. 581 pg/mL, p=0.003) |
| Javorski et al, 2018 [62] | Vitamin D | Cardiac surgery | Cardaic surgery without vasoplegia | Use of ≥2 Vasopressors at high dose with preserved cardiac index | 54, 9.3% | Primary outcome: assess vitamin D deficiency as a risk factor for  vasoplegia after using CPB. | There was no correlation between vitamin D levels and postoperative vasopressor use. |
| Nee et al, 2013 [46] | Troponin-I | Cardiac surgery | Cardiac surgery without vasoplegia | MAP <50mmHg requiring, vasopressors | 86, 19.8% | Primary outcome: influence of troponin on outcomes following cardiac surgery  APC in vasoplegic vs non complicated group | Troponin significantly higher in vasoplegic group at all time points (baseline, during surgery and post operatively) |
| Rosjo et al, 2011 [25] | Troponin-T | Sepsis | Septic not shocked | Not defined | 254, unknown | Primary outcome: assess utility of troponin T for outcome prediction in sepsis and spetic shock  Troponin-T in shocked vs non shocked cohort | Troponin-T higher in patients with septic shock  during the hospitalisation (0.044 vs 0.033lg/L, p= 0.03),  Correlation with noradrenaline dose, r=0.19, p=0.007 . |
| Nee et al, 2013 [46] | Ischaemia modified albumin (IMA) | Cardiac surgery | Cardiac surgery without vasoplegia | MAP <50mmHg requiring, vasopressors | 86, 19.8% | Primary outcome: IMA change with vasoplegia following cardiac surgery  IMA in vasoplegic vs non complicated group | IMA significantly higher in the vasoplegic cohort at all time points measured (p<0.05) |
| Van Lier et al, 2022 [39] | DPP3 | Post cardiac surgery | Cardiac surgery without vasoplegia | MAP <70mmHg not responsive to fluid administration with CI >2.5L/min/m^2 | 208, 15.6% | Primary outcome: to investigate the temporal profile of bio-DPP3  Is DPP3 able to predict prolonged vasopressor dependency | DPP3 levels were not related to post operative vasoplegia |

#

# Appendix 4: Summary information of outcomes

Table of vasoplegia definitions

| **Variable** | **Count** |
| --- | --- |
| **Blood pressure requirements** | |
| No defined blood pressure required | 9 |
| MAP <50mmHg | 4 |
| MAP<60mmHg | 5 |
| MAP<65mmHG | 8 |
| MAP<70mmHG | 4 |
| SBP<90mmHg | 8 |
| SBP<100mmHg | 1 |
| **Vasopressor requirements** | |
| No vasopressor dose | 12 |
| Any vasopressor dose | 18 |
| Noradrenaline >0.1mcg/kg/min | 4 |
| Noradrenaline >0.15mcg/kg/min | 1 |
| Noradrenaline >0.2mcg/kg/min | 1 |
| Noradrenaline >0.25mcg/kg/min | 1 |
| Noradrenaline >0.5mcg/kg/min | 1 |
| More than 2 high dose vasopressors | 1 |
| **Cardiac output monitoring requirements** | |
| No Values Needed | 30 |
| Low Indexed Systemic vascular resistance (SVRI) | 4 |
| Normal Cardiac Index | 5 |
| **Fluid administration requirements** | |
| No fluid administration required | 32 |
| Fluid administration required | 7 |

Table of surrogates of vasoplegia (used in figure 3)

| **Surrogate** | **Count** |
| --- | --- |
| Hypotension | 18 |
| Vasopressor Score | 5 |
| Fluids administered | 1 |
| Need for vasopressors | 21 |
| Noradrenaline equivalent dose | 7 |
| Shock score | 4 |
| SIRS | 4 |
| Vasopressor dose | 9 |
| SVRI | 6 |
| MAP | 8 |

Primary outcome measures:

| **Key phrase** | **Count** |
| --- | --- |
| biomarker trend | 13 |
| biomarker between two groups | 11 |
| biomarker prediction of vasoplegia | 7 |
| biomarker association with vasoplegia | 11 |
| biomarker with outcome | 10 |
| biomarker association with severity of illness | 8 |
| biomarker with aki | 1 |
| role of biomarker in disease | 1 |
| predict vasopressor dependency | 1 |
| biomarker levels for diagnosis | 1 |

Table of outcome measures across all studies

| **Outcome measure** | **Count** |
| --- | --- |
| Sepsis | 3 |
| MI | 3 |
| AKI | 17 |
| MODS score | 4 |
| LOS | 18 |
| Urine output | 2 |
| PF ratio | 1 |
| Shock index | 1 |
| Lactate | 4 |
| APACHE II | 9 |
| Mortality | 24 |
| Sofa score | 14 |
| Mech vent | 9 |
| Stroke | 2 |
| Duration of ventilation | 4 |
| RRT | 5 |
| Platelets | 0 |
| Urine output | 2 |
| Stroke | 2 |
| Sofa score | 14 |

# Appendix 5: search strategy for all databases

## Medline/ Ovid

1. Vasoplegia/

2. (vasopleg* or "vaso-pleg*").ti,ab,kw,kf.

3. 1 or 2

4. Hypotension/

5. (hypotens* or "hypo-tens*").ti,ab,kw,kf.

6. ((low* or loss*) adj3 ("system* vascul* resist*" or "blood pressure*")).ti,ab,kw,kf.

7. 4 or 5 or 6

8. exp Critical Care/

9. (arrest* or code* or critical* or danger* or deadly* or death* or endanger* or excessiv* or fatal* or ICU or ICUs or "intensive care*" or "life-threaten*" or shock* or unusual*).ti,ab,kw,kf.

10. 8 or 9

11. 7 and 10

12. 3 or 11

13. exp Biomarkers/ or exp Risk Factors/ or Protective Factors/ or Risk Assessment/

14. (biomark* or "bio-mark*" or bioparameter* or "bio-parameter*" or marker*).ti,ab,kw,kf.

15. (biologic* adj3 parameter*).ti,ab,kw,kf.

16. ((contribut* or predictiv* or prognos* or protectiv* or risk*) adj3 factor*).ti,ab,kw,kf.

17. (risk* adj3 assess*).ti,ab,kw,kf.

18. (predict* or causalit* or causat* or correlat*).ti,ab,kw,kf.

19. 13 or 14 or 15 or 16 or 17 or 18

20. 12 and 19

21. limit 20 to english language

## Embase

#1 'vasoplegia'/exp

#2 vasopleg*:ti,ab,kw OR 'vaso-pleg*':ti,ab,kw

#3 #1 OR #2

#4 'hypotension'/de

#5 hypotens*:ti,ab,kw OR 'hypo-tens*':ti,ab,kw

#6 ((low* OR loss*) NEAR/3 ('system* vascul* resist*' OR 'blood pressure*')):ti,ab,kw

#7 #4 OR #5 OR #6

#8 'intensive care'/exp

#9 arrest*:ti,ab,kw OR code*:ti,ab,kw OR critical*:ti,ab,kw OR danger*:ti,ab,kw OR deadly*:ti,ab,kw OR death*:ti,ab,kw OR endanger*:ti,ab,kw OR excessiv*:ti,ab,kw OR fatal*:ti,ab,kw OR icu:ti,ab,kw OR icus:ti,ab,kw OR 'intensive care*':ti,ab,kw OR 'life-threaten*':ti,ab,kw OR shock*:ti,ab,kw OR unusual*:ti,ab,kw

#10 #8 OR #9

#11 #7 AND #10

#12 #3 OR #11

#13 'biological marker'/exp OR 'risk factor'/exp OR 'risk assessment'/de OR 'health risk assessment'/de

#14 biomark*:ti,ab,kw OR 'bio-mark*':ti,ab,kw OR bioparameter*:ti,ab,kw OR 'bio-parameter*':ti,ab,kw OR marker*:ti,ab,kw

#15 (biologic* NEAR/3 parameter*):ti,ab,kw

#16 ((contribut* OR predictiv* OR prognos* OR protectiv* OR risk*) NEAR/3 factor*):ti,ab,kw

#17 (risk* NEAR/3 assess*):ti,ab,kw

#18 predict*:ti,ab,kw OR causalit*:ti,ab,kw OR causat*:ti,ab,kw OR correlat*:ti,ab,kw

#19 #13 OR #14 OR #15 OR #16 OR #17 OR #18

#20 #12 AND #19

#21 #12 AND #19 AND [english]/lim

## Web of Science

1 predict* or causalit* or causat* or correlat* (Title) or predict* or causalit* or causat* or correlat* (Abstract) or predict* or causalit* or causat* or correlat* (Author Keywords)

2 risk* near/3 assess* (Title) or risk* near/3 assess* (Abstract) or risk* near/3 assess* (Author Keywords)

3 (contribut* or predictiv* or prognos* or protectiv* or risk*) NEAR/3 factor* (Title) or (contribut* or predictiv* or prognos* or protectiv* or risk*) NEAR/3 factor* (Abstract) or (contribut* or predictiv* or prognos* or protectiv* or risk*) NEAR/3 factor* (Author Keywords)

4 biologic* NEAR/3 parameter* (Title) or biologic* NEAR/3 parameter* (Abstract) or biologic* NEAR/3 parameter* (Author Keywords)

5 biomark* or "bio-mark*" or bioparameter* or "bio-parameter*" or marker* (Title) or biomark* or "bio-mark*" or bioparameter* or "bio-parameter*" or marker* (Abstract) or biomark* or "bio-mark*" or bioparameter* or "bio-parameter*" or marker* (Author Keywords)

6 #1 OR #2 OR #3 OR #4 OR #5

7 vasopleg* or "vaso-pleg*" (Title) or vasopleg* or "vaso-pleg*" (Abstract) or vasopleg* or "vaso-pleg*" (Author Keywords)

8 hypotens* or "hypo-tens*" (Title) or hypotens* or "hypo-tens*" (Abstract) or hypotens* or "hypo-tens*" (Author Keywords)

9 (low* or loss*) NEAR/3 ("system* vascul* resist*" or "blood pressure*") (Title) or (low* or loss*) NEAR/3 ("system* vascul* resist*" or "blood pressure*") (Abstract) or (low* or loss*) NEAR/3 ("system* vascul* resist*" or "blood pressure*") (Author Keywords)

10 #8 OR #9

11 arrest* or code* or critical* or danger* or deadly* or death* or endanger* or excessiv* or fatal* or ICU or ICUs or "intensive care*" or "life-threaten*" or shock* or unusual* (Title) or arrest* or code* or critical* or danger* or deadly* or death* or endanger* or excessiv* or fatal* or ICU or ICUs or "intensive care*" or "life-threaten*" or shock* or unusual* (Abstract) or arrest* or code* or critical* or danger* or deadly* or death* or endanger* or excessiv* or fatal* or ICU or ICUs or "intensive care*" or "life-threaten*" or shock* or unusual* (Author Keywords)

12 #10 AND #11

13 #7 OR #12

14 #6 AND #13

## Cochrane

#1 MeSH descriptor: [Vasoplegia] this term only

#2 (vasopleg* or "vaso-plegia" or "vaso-plegic"):ti,ab,kw (Word variations have been searched)

#3 #1 OR #2

#4 MeSH descriptor: [Hypotension] this term only

#5 (hypotens* or "hypo-tension" or "hypo-tensive"):ti,ab,kw (Word variations have been searched)

#6 ((low* or loss*) NEAR/3 ("systemic vascular resistance" or "blood pressure")):ti,ab,kw (Word variations have been searched)

#7 #4 OR #5 OR #6

#8 MeSH descriptor: [Critical Care] explode all trees

#9 (arrest* or code* or critical* or danger* or deadly* or death* or endanger* or excessiv* or fatal* or ICU or ICUs or "intensive care" or "life-threatening" or shock* or unusual*):ti,ab,kw (Word variations have been searched)

#10 #8 OR #9

#11 #7 AND #10

#12 #3 OR #11

#13 MeSH descriptor: [Biomarkers] explode all trees

#14 MeSH descriptor: [Risk Factors] explode all trees

#15 MeSH descriptor: [Protective Factors] this term only

#16 MeSH descriptor: [Risk Assessment] this term only

#17 (biomark* or "bio-marker" or "bio-markers" or bioparameter* or "bio-parameter" or "bio-parameters" or maker*):ti,ab,kw (Word variations have been searched)

#18 (biologic* NEAR/3 parameter*):ti,ab,kw (Word variations have been searched)

#19 ((contribut* or predictiv* or prognos* or protectiv* or risk*) NEAR/3 factor*):ti,ab,kw (Word variations have been searched)

#20 (predict* or causalit* or causal* or correlat*):ti,ab,kw (Word variations have been searched)

#21 #13 OR #14 OR #15 OR #16 OR #17 OR #18 OR #19 OR #20

#22 #12 AND #21

# Appendix 6: Prisma checklist

| **Section and Topic** | **Item #** | **Checklist item** | **Location where item is reported** |
| --- | --- | --- | --- |
| **TITLE** | | |  |
| Title | 1 | Identify the report as a systematic review. | 1 |
| **ABSTRACT** | | |  |
| Abstract | 2 | See the PRISMA 2020 for Abstracts checklist. | 1 |
| **INTRODUCTION** | | |  |
| Rationale | 3 | Describe the rationale for the review in the context of existing knowledge. | 3 |
| Objectives | 4 | Provide an explicit statement of the objective(s) or question(s) the review addresses. | 4 |
| **METHODS** | | |  |
| Eligibility criteria | 5 | Specify the inclusion and exclusion criteria for the review and how studies were grouped for the syntheses. | 5 |
| Information sources | 6 | Specify all databases, registers, websites, organisations, reference lists and other sources searched or consulted to identify studies. Specify the date when each source was last searched or consulted. | 5 |
| Search strategy | 7 | Present the full search strategies for all databases, registers and websites, including any filters and limits used. | Appendix 5 |
| Selection process | 8 | Specify the methods used to decide whether a study met the inclusion criteria of the review, including how many reviewers screened each record and each report retrieved, whether they worked independently, and if applicable, details of automation tools used in the process. | 5 + 6 |
| Data collection process | 9 | Specify the methods used to collect data from reports, including how many reviewers collected data from each report, whether they worked independently, any processes for obtaining or confirming data from study investigators, and if applicable, details of automation tools used in the process. | 6 |
| Data items | 10a | List and define all outcomes for which data were sought. Specify whether all results that were compatible with each outcome domain in each study were sought (e.g. for all measures, time points, analyses), and if not, the methods used to decide which results to collect. | 5+ 6 |
|  | 10b | List and define all other variables for which data were sought (e.g. participant and intervention characteristics, funding sources). Describe any assumptions made about any missing or unclear information. | 5+ 6 + 7 |
| Study risk of bias assessment | 11 | Specify the methods used to assess risk of bias in the included studies, including details of the tool(s) used, how many reviewers assessed each study and whether they worked independently, and if applicable, details of automation tools used in the process. | 6 |
| Effect measures | 12 | Specify for each outcome the effect measure(s) (e.g. risk ratio, mean difference) used in the synthesis or presentation of results. | 7 |
| Synthesis methods | 13a | Describe the processes used to decide which studies were eligible for each synthesis (e.g. tabulating the study intervention characteristics and comparing against the planned groups for each synthesis (item #5)). | 6 |
|  | 13b | Describe any methods required to prepare the data for presentation or synthesis, such as handling of missing summary statistics, or data conversions. | 6 |
|  | 13c | Describe any methods used to tabulate or visually display results of individual studies and syntheses. | 6 |
|  | 13d | Describe any methods used to synthesize results and provide a rationale for the choice(s). If meta-analysis was performed, describe the model(s), method(s) to identify the presence and extent of statistical heterogeneity, and software package(s) used. | 6 +7 |
|  | 13e | Describe any methods used to explore possible causes of heterogeneity among study results (e.g. subgroup analysis, meta-regression). | 6 +7 |
|  | 13f | Describe any sensitivity analyses conducted to assess robustness of the synthesized results. | 6 +7 |
| Reporting bias assessment | 14 | Describe any methods used to assess risk of bias due to missing results in a synthesis (arising from reporting biases). | 6 +7 |
| Certainty assessment | 15 | Describe any methods used to assess certainty (or confidence) in the body of evidence for an outcome. | 6 +7 |
| **RESULTS** | | |  |
| Study selection | 16a | Describe the results of the search and selection process, from the number of records identified in the search to the number of studies included in the review, ideally using a flow diagram. | Appendix 1 |
|  | 16b | Cite studies that might appear to meet the inclusion criteria, but which were excluded, and explain why they were excluded. | Appendix 1 |
| Study characteristics | 17 | Cite each included study and present its characteristics. | 8+ 9+ 10 + 12 + Appendix 3 |
| Risk of bias in studies | 18 | Present assessments of risk of bias for each included study. | 9 |
| Results of individual studies | 19 | For all outcomes, present, for each study: (a) summary statistics for each group (where appropriate) and (b) an effect estimate and its precision (e.g. confidence/credible interval), ideally using structured tables or plots. | Appendix 4 |
| Results of syntheses | 20a | For each synthesis, briefly summarise the characteristics and risk of bias among contributing studies. | 9 |
|  | 20b | Present results of all statistical syntheses conducted. If meta-analysis was done, present for each the summary estimate and its precision (e.g. confidence/credible interval) and measures of statistical heterogeneity. If comparing groups, describe the direction of the effect. | N/A |
|  | 20c | Present results of all investigations of possible causes of heterogeneity among study results. | N/A |
|  | 20d | Present results of all sensitivity analyses conducted to assess the robustness of the synthesized results. | N/A |
| Reporting biases | 21 | Present assessments of risk of bias due to missing results (arising from reporting biases) for each synthesis assessed. | N/A |
| Certainty of evidence | 22 | Present assessments of certainty (or confidence) in the body of evidence for each outcome assessed. | N/A |
| **DISCUSSION** | | |  |
| Discussion | 23a | Provide a general interpretation of the results in the context of other evidence. | 16 + 18 |
|  | 23b | Discuss any limitations of the evidence included in the review. | 16 +17 |
|  | 23c | Discuss any limitations of the review processes used. | 16 +17 |
|  | 23d | Discuss implications of the results for practice, policy, and future research. | 19 |
| **OTHER INFORMATION** | | |  |
| Registration and protocol | 24a | Provide registration information for the review, including register name and registration number, or state that the review was not registered. | 5 |
|  | 24b | Indicate where the review protocol can be accessed, or state that a protocol was not prepared. | 5 |
|  | 24c | Describe and explain any amendments to information provided at registration or in the protocol. | N/A |
| Support | 25 | Describe sources of financial or non-financial support for the review, and the role of the funders or sponsors in the review. | 22 |
| Competing interests | 26 | Declare any competing interests of review authors. | 22 |
| Availability of data, code and other materials | 27 | Report which of the following are publicly available and where they can be found: template data collection forms; data extracted from included studies; data used for all analyses; analytic code; any other materials used in the review. | N/A |
